# Immunological Regulation of Vascular Inflammation During Cancer Metastasis

**DOI:** 10.3389/fimmu.2019.01984

**Published:** 2019-08-21

**Authors:** Sheri A. C. McDowell, Daniela F. Quail

**Affiliations:** ^1^Department of Physiology, Faculty of Medicine, McGill University, Montreal, QC, Canada; ^2^Rosalind and Morris Goodman Cancer Research Centre, McGill University, Montreal, QC, Canada; ^3^Division of Experimental Medicine, Department of Medicine, McGill University, Montreal, QC, Canada

**Keywords:** metastasis, microenvironment, vascular inflammation, innate immunity, endothelial adhesions

## Abstract

Metastasis is the predominant cause of cancer-related mortality, despite being a highly inefficient process overall. The vasculature is the gatekeeper for tumor cell seeding within the secondary tissue microenvironment—the rate limiting step of the metastatic cascade. Therefore, factors that regulate vascular physiology dramatically influence cancer outcomes. There are a myriad of physiologic circumstances that not only influence the intrinsic capacity of tumor cells to cross the endothelial barrier, but also that regulate vascular inflammation and barrier integrity to enable extravasation into the metastatic niche. These processes are highly dependent on inflammatory cues largely initiated by the innate immune compartment, that are meant to help re-establish tissue homeostasis, but instead become hijacked by cancer cells. Here, we discuss the scientific advances in understanding the interactions between innate immune cells and the endothelium, describe their influence on cancer metastasis, and evaluate potential therapeutic interventions for the alleviation of metastatic disease. By triangulating the relationship between immune cells, endothelial cells, and tumor cells, we will gain greater insight into how to impede the metastatic process by focusing on its most vulnerable phases, thereby reducing metastatic spread and cancer-related mortality.

## Introduction

Metastasis is a process through which primary tumor cells spread to secondary organs, and is the leading cause of cancer-related mortality. The metastatic process is composed of a number of sequential steps, each with varying levels of efficiency that together dictate whether successful metastases will form (1). Initially, cancer cells from a primary tumor acquire the capacity to invade into adjacent tissue and intravasate into the blood circulatory or lymphatic system. Within the circulation, cancer cells must survive in suspension and interact with the endothelium in order to extravasate into the secondary tissue parenchyma. In parallel, the endothelium becomes primed to allow cancer cells to transmigrate, and the pre-metastatic niche evolves into a fertile soil equipped to nurture metastatic cells. Upon arrival, cancer cells quickly adapt to the foreign niche, to enable their colonization and outgrowth within the new microenvironment. Each of these stages requires cancer cells to exhibit remarkable plasticity, allowing them to adapt to continuous changes and unfamiliar stimuli that are encountered within their surroundings.

The overall process of metastasis is highly inefficient, and early kinetic studies using experimental metastasis models have shown that the efficiency of each individual step of the metastatic cascade differs dramatically (2, 3). Early steps, such as local invasion and survival within the circulation, are very efficient; however, later stages that take place within the secondary niche are relatively inefficient. In cancer patients, although the frequency of circulating tumor cells is an independent predictor of overall survival (4), some patients with circulating tumor cells within their blood may never develop metastatic disease. This suggests that metastatic potential is partially influenced by the ability of circulating tumor cells to access the metastatic microenvironment (5–9). Therefore, understanding how the vasculature acts as the gatekeeper for metastatic disease is critical to limit disease progression.

The role of tumor cell-mediated mechanisms of extravasation during metastasis has been covered by several excellent reviews (10–12). Here, we discuss immune-mediated mechanisms of vascular physiology that influence extravasation efficiency, with a focus on innate immune mechanisms of vascular inflammation and metastasis. We first discuss how the structure of the endothelium mediates vascular inflammation (including permeability of the endothelium, and transmigration of leukocytes), and how chronic inflammatory conditions that have direct ties to cancer (e.g., obesity, smoking tobacco) can exacerbate these effects. We then discuss the role of vascular inflammation during cancer metastasis, and how cancer cells can hijack innate immune processes to enhance their metastatic behavior. Finally, we discuss how mechanisms of vascular inflammation can be targeted as a preventative approach for metastatic disease.

## Structure and Function of the Endothelial Barrier During Vascular Inflammation

**Figure 1:**
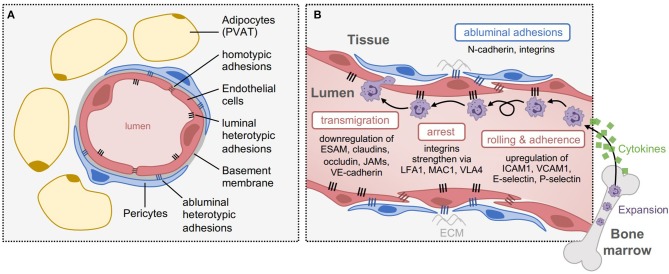
Structure and function of the endothelium during vascular inflammation. **(A)** Structure of blood capillaries, with surrounding perivascular adipose tissue (PVAT). The lumen is formed by 1-2 endothelial cells that are sealed by homotypic junctional adhesions, including tight junctions and adherens junctions. The endothelial cells are bound to a specialized basement membrane, and enveloped with pericytes once mature. On their luminal side, endothelial cells express heterotypic adhesions that assist with cell attachment within the periphery, and on their abluminal side, they express distinct heterotypic adhesions (e.g., N-cadherin and integrins) that facilitate pericyte coverage and attachment to the extracellular matrix (ECM). **(B)** Adhesions involved in different steps of leukocyte transmigration. In response to inflammation, cytokines are released to cause expansion and recruitment of leukocytes from the periphery, such as neutrophils from the bone marrow. Upon arrival at the inflamed tissue, leukocytes roll and adhere to the endothelium through luminal adhesion proteins including upregulation of E-selectin, P-selectin, ICAM1, and VCAM1. To induce their arrest, integrins strengthen these interactions through interactions with LFA1, MAC1, and VLA4, which are expressed by leukocytes. Once arrested, transmigration can occur through endothelial junctions, by downregulating homotypic adhesions such as ESAM, claudins, occludin, JAMs, and VE-cadherin.

Blood vessels function as boundaries between blood and tissue, by regulating permeability, blood fluidity, and the flow of cells and other substances between the vascular system and tissues throughout the body. Generally, mature blood vessels consist of a monolayer of endothelial cells connected to one another through distinct junctional boundaries, which are further wrapped by pericytes or vascular smooth muscle cells that maintain structural support and integrity, and in most tissues, are enveloped by adipose tissue (Figure 1A). However, the intricacies of vascular endothelia architecture vary between different organs and vascular beds, resulting in differences in mechanisms of leukocyte trafficking during inflammation. For example, in the skin, muscle and mesentery leukocytes typically exit the blood in postcapillary venules, while in the lung and liver leukocytes exit the blood in the microvasculature, and in lymphoid organs leukocytes exit the blood in high endothelial venules (HEV); all of these endothelia have different structures, functions, and blood flow dynamics (13). Furthermore, there are differences in leukocyte trafficking between different leukocyte subsets. For example, innate immune cells are structurally and functionally different from adaptive immune cells and thus the cellular and molecular mechanisms of recruitment and extravasation are distinct; this review will focus on the extravasation of innate immune cells—the first responders to inflammatory stimuli.

Under inflammatory contexts, the endothelium becomes activated to facilitate leukocyte recruitment into the affected tissue through a process called vascular inflammation (14, 15). During vascular inflammation, luminal endothelial adhesion proteins are upregulated to enhance leukocyte rolling, arrest and adherence to the endothelium, even when exposed to high shear stress (16), and in parallel, endothelial junctional adhesions are downregulated to enable leukocyte transmigration (17). It has been proposed that cancer cells mimic leukocyte transmigration to facilitate their extravasation into tissues, therefore, insights that are gained from leukocyte dynamics with the endothelium may be relevant to cancer metastasis.

Several canonical adhesion proteins regulate transmigration of cells across the endothelium (Figure 1B). Heterotypic endothelial adhesions regulate interactions between endothelial cells and their surrounding environment. On the luminal side, this includes interactions with circulating immune cells, which need to arrest and adhere to the endothelium prior to transmigration. These heterotypic interactions are mediated by a distinct set of luminal transmembrane adhesive proteins, such as selectins (e.g., E-selectin, P-selectin; leukocyte rolling) and Immunoglobulin (Ig)-like cell adhesion molecules (e.g., ICAM1, VCAM1; leukocyte arrest, firm adhesion, and crawling) (17). On the abluminal side, endothelial adhesions mediate interactions with pericytes and the extracellular matrix, such as neural (N)-cadherin, which regulates pericyte coverage and vessel maturity. By contrast, homotypic endothelial adhesions primarily function to regulate barrier integrity of the endothelium and vascular permeability, and are thus composed of proteins involved in tight junctions (e.g., junctional adhesion molecules (JAMs), claudins, and occludin) and adherens junctions (e.g., vascular endothelial (VE)-cadherin, which associates with the intracellular β-catenin protein) between endothelial cells (18). These adhesions play an important role specifically during the process of cellular transmigration. Collectively, both heterotypic and homotypic adhesion proteins act as gatekeepers of tissue homeostasis, and therefore, the plasticity of endothelial adhesion expression is essential to this phenotype.

### Leukocyte Rolling, Adherence, and Transmigration Across the Activated Endothelium

Patrolling leukocytes move through blood vessels in a passive manner based on simple flow dynamics. However, under inflammatory conditions, leukocytes are attracted to specific tissues through cytokines that are produced in response to pathogen exposure and/or tissue damage (19). Once leukocytes arrive, infiltration into tissues is first initiated by rolling along on the activated endothelium, which is frequently mediated by selectin-based interactions between immune and endothelial cells. Endothelial cells express selectin proteins, such as P- and E-selectin, along with ligands for L-selectin (L-selectin is expressed on naïve leukocytes prior to activation), while leukocytes express glycosylated selectin ligands, such as P-selectin Glycoprotein Ligand-1 (PSGL-1; constitutively expressed by neutrophils) and E-selectin ligand-1 (ESL-1) (20, 21). Selectin-mediated rolling activates leukocytes by facilitating interactions with inflammatory chemokines from the activated endothelium such as interleukin-8 (IL8) (22) and platelet-activating factor (PAF) (23), which bind to chemokine receptors on leukocytes to initiate a signaling cascade resulting in the activation of integrins (20, 24, 25). Integrin-mediated signaling via lymphocyte function-associated antigen 1 (LFA1; expressed by all leukocytes), macrophage antigen 1 (MAC1; expressed by monocytes/macrophages), and very late antigen 4 (VLA4; expressed by lymphocytes and monocytes) increases the affinity of immune cells for the endothelium, allowing for more firm and stable adherence, in preparation for subsequent transmigration across the endothelial barrier (26). In addition, leukocytes may crawl toward suitable emigration sites prior to extravasation, depending on the inflammatory phenotype of the endothelium, as well as the activation state and type of leukocyte (27). For example, intravital videomicroscopy of murine postcapillary venules has shown that following adhesion to the endothelium, neutrophils crawl intraluminally to sites of extravasation prior to transmigration (27). Thus, luminal endothelial adhesion proteins are the first line of regulation of peripheral cell infiltration into tissues.

Of note, platelets (cell fragments derived from megakaryocytes from bone marrow) also play a role in the extravasation of leukocytes. They typically function to form blood clots, but more recently have been shown to play a role in vascular inflammation (28). In addition to being able to interact with both immune and endothelial cells, a novel role for platelets in efficiently directing neutrophils and inflammatory monocytes to sites of extravasation has been identified, whereby platelets interact with endothelial cells and arrest neutrophils upon initiation of an inflammatory stimulus (29). This interaction then mobilizes inflammatory monocytes to these specific locations, facilitating the successful extravasation of both neutrophils and inflammatory monocytes into the tissue parenchyma.

Once leukocytes establish tight adhesions at endothelial junctions, they begin the process of extravasation known as diapedesis (30). Diapedesis most often occurs through a paracellular pathway (i.e., in between cells of the endothelial barrier). This is mainly regulated by changes in vascular permeability via tight junctions, including downregulation of endothelial cell-selective adhesion molecule (ESAM) (31), and tight JAMs, JAMA, JAMB, and JAMC (30, 32, 33). Less frequently, leukocytes may also transmigrate through the transcellular pathway (i.e., through the endothelial cell body), which is dependent on the formation of transmigratory cup-like projections that are enriched for ICAM1 and VCAM1 (34). Given that vascular inflammation usually requires a more rapid response rate, regulation of adhesion molecule expression is usually done at the post-translational level. For example, this can be achieved via proteolytic cleavage induced by innate immune cells within the microenvironment, such as neutrophil-derived neutrophil elastase (NE) (35). Other methods of regulation include the post-translational modification of integrins, along with changes in the storage and release of selectins to the cell membrane, specifically P-selectin. P-selectin, is stored in Weibel-Palade bodies (WPB) in endothelial cells and becomes recruited to the cell membrane upon inflammatory signals (36). Cell adhesion molecules, such as ICAM1 and VCAM1, can be regulated through changes in expression. For example IL1β-, tumor necrosis factor-α (TNFα)-, or lipopolysaccharide (LPS)-stimulated endothelial cells can induce expression of VCAM1 and enhance expression of ICAM1 (37). It is important to note that extravasation not only mediates leukocyte recruitment to sites of inflammation, but also regulates leukocyte phenotype. This enables leukocytes to be better equipped to pursue further migration and specific immune functions, for example, increased survival and pathogen-killing activities (38).

Once leukocytes permeate the endothelial cell barrier they encounter the endothelial basement membrane network made up of protein laminins (e.g., laminin-8 and laminin-10), collagen type IV, nidogens, and heparan sulfate proteoglycan perlecan (39). In the majority of venules, leukocytes will also encounter the pericyte sheath and perivascular tissue. Neutrophil migration through the basement membrane and pericyte sheath has been shown to occur at sites with low extracellular matrix protein accumulation, specifically laminin-10, collagen IV and nidogen-2, and between neighboring pericytes in murine cremasteric venules (40). Similarly, monocytes have been shown to use comparable methods to cross the basement membrane in CCL2-stimulated murine cremaster muscles (41).

Taken together, each of these factors that regulate transmigration of cells across the endothelium may have relevance to cancer, if similar mechanisms are used by tumor cells.

### Factors That Regulate Vascular Inflammation and Barrier Integrity

There are numerous factors that regulate endothelial adhesions, and as a consequence influence vascular inflammation and permeability. Many of these factors play a crucial role in physiologic oxidative functions of innate immunity, to facilitate subsequent amplification of inflammatory safeguards when pathogens or tissue damage are detected. For example, activated neutrophils produce reactive oxygen species (ROS) during vascular inflammation which can have effects on the surrounding tissue microenvironment, notably endothelial junctional integrity (15). Activated porcine neutrophils cultured with endothelial monolayers have been shown to enhance endothelial permeability by altering phosphorylation of VE-cadherin and β-catenin, resulting in conformational changes to the adherens junctions that disrupt endothelial barrier function (42). Of note, the VE-cadherin-catenin complex in adherens junctions can be regulated by ROS via induction of phosphorylation which promotes junctional disassembly (43), and is required for neutrophil transendothelial migration (44), highlighting an important link between neutrophils, ROS, and vascular permeability. Other innate immune cells such as macrophages can be a major source of vascular endothelial growth factor A (VEGFA) within the microenvironment, which can also induce oxidative stress and vascular permeability by phosphorylating VE-cadherin (45) or causing its endocytosis (46). In mouse models of sterile injury, leukotrienes have also been shown to act on neutrophils to induce their release of NE to cleave JAM-C (35). Interestingly, intravital microscopy has shown that neutrophil communication with the endothelium in this manner can also enable reverse transmigration of neutrophils from local tissues back into the peripheral circulation (35, 47), however, it is unclear if this process serves to resolve local inflammation, or to amplify systemic immunological responses. Taken together, while these inflammatory responses function as an acute protective mechanism for the host, chronic vascular inflammation can be detrimental.

Indeed, there are numerous pathological conditions that can aberrantly weaken the endothelial barrier, which have strong ties to cancer. This can leave the host prone to immune exhaustion, disruption of tissue homeostasis, edema, or nutrient imbalance, and ultimately may modify the ability of cancer cells to extravasate into secondary tissues. For example, obesity is a chronic inflammatory condition that is linked to numerous co-morbidities that affect the vascular system, such as hypertension, coronary artery disease, and stroke, and is associated with enhanced cancer incidence (48) and mortality (49). In fact, obesity is thought to be responsible for up to 20% of cancer-related deaths in adults (49), making it a leading risk factor for cancer mortality. Indeed, there exists an intimate relationship between adipose tissue and the vascular system, both anatomically and functionally, as the majority of blood vessels are enclosed by perivascular adipose tissue (PVAT), which plays a role in guiding vascular function and homeostasis by releasing a myriad of bioactive adipokines and cytokines (50). Under normal physiologic conditions, PVAT secretes anti-inflammatory factors and hormones, such as adiponectin, which have protective effects on the cardiovascular system (51). However, during weight gain, adipocytes within PVAT exhibit impaired differentiation and increased expression of pro-inflammatory cytokines, such as interleukin-6 (IL6), IL8, and monocyte chemoattractant protein-1 (MCP1) (52), leptin production (53, 54), and oxidative stress (55) which lead to vascular dysfunction.

In addition to the direct effects of PVAT on adjacent endothelium, obesity-associated adipose tissue can also have systemic effects that influence vascular function. For example, in lung (one of the most frequent sites of cancer metastasis), mouse models have shown that obesity impairs vascular homeostasis when adiponectin levels drop, characterized by an increase in the expression of luminal adhesions including ICAM1, VCAM1, and E-selectin, and a decrease in endothelial adhesions such as VE-cadherin (56). These changes increase neutrophil influx into the lung parenchyma and enhance susceptibility to lung injury by LPS (a side effect of the leaky gut epithelium), which can be attenuated by hydrodynamic adiponectin gene delivery (56). In humans, obesity is similarly associated with oxidative stress and endothelial activation, as assessed by increased plasma levels of oxidized low-density lipoprotein, C-reactive protein, and soluble forms of ICAM1 and E-selectin (57, 58). In porcine models of diet-induced obesity, high-fat diet is associated with elevated superoxide species, nitrotyrosine and NADPH-oxidase subunits in the coronary endothelium, in concordance with enhanced myocardial microvascular permeability prior to the development of insulin resistance (59). These data suggest that oxidative stress and vascular dysfunction may precede the chronic inflammatory effects of obesity that present with the onset of metabolic syndrome. Given the association between obesity and cancer mortality, these findings raise the possibility that obesity-associated vascular inflammation may facilitate tumor cell transendothelial migration, akin to its effects on leukocytes.

Surpassing the effects of obesity on cancer mortality risk, cigarette smoking remains the leading risk factor for lung cancer, and remarkably, is responsible for ~22% of all cancer-related deaths (60). In addition to cancer, smoking tobacco is associated with numerous cardiovascular conditions including atherosclerosis, heart disease, and acute lung injury (61, 62), which is not surprising given the highly vascularized nature of lung tissue. Similar to obesity, smoking causes profound lung inflammation (e.g., increased IL10 and TNFα production, and accumulation of neutrophils and alternatively-activated macrophages), and susceptibility to LPS-induced acute lung injury (63), which together underlie vascular inflammation. In addition to direct effects on the lung capillaries, numerous studies have demonstrated that exposure to cigarette smoke is also associated with a reduction of vascular function in many tissues in the body, linked to aberrant nitric oxide (NO) production (64), an increase in inflammatory markers (e.g., TNFα) (65), and local recruitment of leukocytes to the endothelium (66). In fact, there is even evidence that certain chemical components of cigarettes can weaken endothelial junctions of the blood-brain barrier (67). This may in part explain the high propensity of lung cancer patients to exhibit metastatic disease to the brain compared to other primary malignancies, although this has not been formally tested. Given the causal connection between smoking tobacco and lung cancer incidence and mortality, the effects of smoking on vascular function may have multifaceted effects on cancer progression.

Taken together, chronic inflammatory conditions can mediate changes in endothelial cell homeostasis and alter vascular permeability, much in the same way that an acute inflammatory stimulus does. These conditions (and others) share a common theme of affecting vascular permeability through aberrant production of inflammatory mediators, and notably through enhanced oxidative stress. How these disease states and their corresponding effects on the vascular system affect metastatic efficiency, particularly during transmigration of tumor cells across the endothelial barrier, is a key question that remains largely unexplored.

## Vascular Inflammation During Cancer Metastasis: Influence of the Innate Immune System

**Figure 2:**
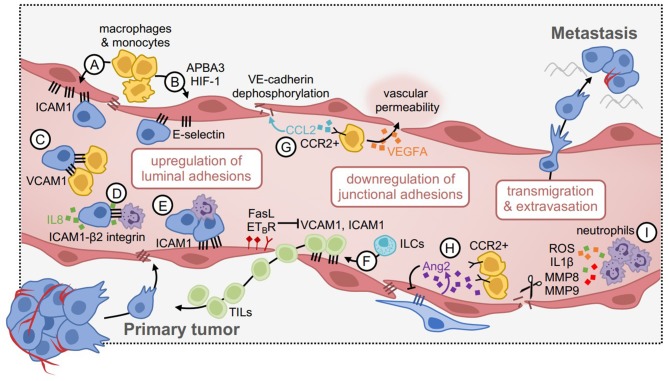
Vascular inflammation during tumor cell extravasation and metastasis. **(A)** Inflammatory macrophages and monocytes induce the expression of ICAM1 and **(B)** E-selectin via APBA3-HIF1 signaling in endothelial cells. This enhances tumor cell attachment. **(C)** Tumor cells mimic the endothelium by upregulating VCAM1, which tethers them to β1 integrin-expressing macrophages and enhances metastatic seeding. **(D)** Similarly, tumor cells can upregulate ICAM1, and tethers to β2-integrin expressing neutrophils. This interaction is mediated by IL8 produced by tumor cells, which promotes neutrophil recruitment. **(E)** Neutrophil-tumor cell clusters enhance attachment to the endothelium via ICAM1 under flow conditions. **(F)** Some positive effects of vascular inflammation include enhanced recruitment of tumor-infiltrating lymphocytes (TILs), through upregulation of VCAM1 and ICAM1. This pathway can be negatively regulated, for example via FasL and ET_B_R, such that blocking these factors can improve TIL delivery to tumors. **(G)** CCR2+ monocytes are attracted to the metastatic niche in response to CCL2 expressed by tumor cells, where they produce VEGFA to increase vascular permeability. CCL2 can also act directly on the endothelium by dephosphorylating VE-cadherin, disrupting junctional integrity, and increasing tumor cell transmigration. **(H)** Angiopoietin 2 (Ang2) expression by endothelial cells reduces pericyte coverage and promotes the recruitment of CCR2+ monocytes, which promote permeability. **(I)** Neutrophils produce proteolytic and inflammatory factors (e.g., ROS, IL1β, MMP8, MMP9) to disrupt endothelial junctions and increase vascular permeability in cancer models.

It is well-accepted that inflammation can strongly impact tumor progression (68, 69). Similar to the consequences of vascular inflammation on permeability of the endothelium and leukocyte transmigration, it has been proposed that metastatic tumor cells can mimic leukocyte behavior and exploit the inflammatory effects of cancer to assist their spread to secondary organs (70) (Figure 2). This is achieved through upregulation of heterotypic adhesions on the endothelium to facilitate tumor cell rolling and transendothelial migration, and in parallel, weakening of vascular integrity to facilitate tumor cell crossing. Immune cells that are activated toward a pro-tumorigenic phenotype participate in both of these processes, by secreting pro-inflammatory factors that activate the endothelium. These immune cells are recruited to the perivascular microenvironment through tumor-derived factors, or in response to other underlying inflammatory conditions. Therefore, a comprehensive understanding of the mechanisms that mediate these processes may be useful to develop therapeutics to prevent metastatic progression.

### Vascular Trapping, Luminal Adhesions and Tumor Cell Rolling

Numerous studies have investigated tumor cell extravasation using *in vivo* imaging techniques (71–73). As leukocytes are relatively small, they can comfortably roll along blood vessels during leukocyte trafficking. However, tumor cells can be much larger in diameter and may not be able to move through blood vessels as easily. Studies have investigated the relative contribution of physical trapping due to size constraints versus the distinct adhesion of tumor cells during shear-resistant arrest. Intravital videomicroscopy in mice has demonstrated that fluorescently labeled Chinese Hamster Ovary (CHO-K1) cells initially arrest in liver sinusoids following injection into the mesenteric vein due to mechanical trapping (72). Similarly, mechanical trapping and tumor cell arrest has been observed in melanoma and sarcoma models when vessel diameter was less than tumor cell diameter (71, 73). However, studies have also shown that tumor cells can arrest in capillaries in the absence of physical trapping by forming vascular adhesions. For example, colon cancer cells injected into rats were observed to arrest in microvessels even when vessel diameter was greater than tumor cell diameter (74). Similarly, both human HT-29 and murine CC531 colon cancer cells injected intraarterially into rats were shown to adhere to sinusoidal capillaries that were larger in diameter than the tumor cells themselves (75). Tumor cells may therefore become trapped in capillaries due to size-restriction, or form adhesions to the endothelium in the absence of mechanical trapping.

Once tumor cells are trapped in or adhere to blood capillaries, they must cross endothelial barriers. To achieve this, tumor cells utilize many of the same pathways that mediate leukocyte transmigration under inflammatory conditions, such as selectins and cell adhesion molecules (76, 77). Selectin-mediated rolling of tumor cells has been described, but appears to be less common than selectin-mediated leukocyte rolling prior to firm adhesion and extravasation. Nonetheless, rolling of human bone-metastatic prostate tumor cells has been reported, and relies on E-selectin expression on bone marrow endothelial cells and the complimentary expression of cognate ligands on the tumor cells (78). E-selectin-dependent tumor cell rolling on the endothelium following TNF activation has also been described for breast and colon cancer cells (79). However, breast and prostate cancer cells have been shown to express Thomsen-Friedenreich antigen to mediate their arrest on the endothelium via interactions with galectin-3 (80). Furthermore, prostate cancer cell expression of selectin ligands does not correlate with selectin-mediated adhesion to the endothelium (81). This suggests that tumor cells may express selectin ligands, but may not necessarily use them for initial tethering and rolling on the endothelium. Thus, whether selectin-mediated adhesions are requisite for tumor cell binding to the endothelium and extravasation remains unclear.

Tumor cells may also utilize mechanisms initiated by innate immune cells within the microenvironment, which can activate vascular inflammation. For example, macrophages and monocytes have been shown to influence endothelial activation by regulating the expression of luminal adhesions such as ICAM1 (82, 83). In syngeneic melanoma models, glycolytic macrophages upregulate the expression of E-selectin on the endothelium through HIF-1 and its activator APBA3, such that APBA3 depletion in monocytes reverses this effect in association with reduced metastasis to lung (84). In breast cancer models, tumor cells mimic the inflammatory state of the endothelium via endogenous expression of VCAM1, which tethers them to macrophages expressing α4β1 integrin that promote metastasis to the lung (85). Surprisingly, VCAM1 depletion in tumor cells had no influence on the ability of cancer cells to cross the endothelium, rather, this vasculogenic mimicry phenotype enhanced the ability of cancer cells to colonize and remain viable within the secondary niche. This is consistent with reports that the perivascular space acts as a specialized reservoir for cancer stem cell viability (86), and also regulates dormancy in the metastatic setting (87). It is thus conceivable that the viability and/or growth of cancer cells could be influenced by adhesion factors expressed by the adjacent endothelium within this niche, in addition to the capacity for transmigration.

As with vascular permeability, neutrophil-supplied factors can also influence the expression of luminal adhesions that facilitate tumor cell rolling and attachment to the endothelium as they travel through the circulation. For example, *in vitro* microfluidic models of the human microvasculature have shown that LPS-stimulated neutrophils and melanoma cells form aggregates under flow conditions, and arrest on the endothelium in part due to neutrophil-endothelial cell interactions via ICAM1. This heterotypic clustering mechanism could be reversed by blocking ICAM1 on vessels or tumor cells, however, endothelial-specific ICAM1 blockade was much more potent, suggesting that ICAM1 enables tumor cell attachment through both direct and indirect mechanisms (88). Similarly, in mouse models of melanoma, melanoma-specific expression of ICAM1 facilitated tumor cell-neutrophil interactions via β2 integrin on neutrophils, which facilitated attachment to the endothelium in the secondary lung microenvironment (89). This was dependent on IL8-secretion by melanoma cells, a potent neutrophil chemokine, indicating that tumor cells manipulate their environment to support their own progression. Taken together, heterotypic endothelial adhesions appear not only to enable tumor cell adherence to the endothelium during metastasis, but also enable tumor cell tethering to innate immune cells within the microenvironment which further support transmigration.

Although luminal adhesions can facilitate tumor cell extravasation during metastasis, they can also improve anti-tumor immunity by facilitating immune cell access to the tumor niche. For example, in a mouse model of melanoma, NKp46+ innate lymphoid cells (ILCs) upregulate vascular adhesions such as ICAM1 and VCAM1, which facilitate the infiltration of additional immune cells with anti-tumor functions (90). In mice lacking NKp46+ ILCs, this phenotype was reversed. In mouse models of ovarian cancer, overexpression of the endothelin B receptor (ET_B_R) negatively regulates ICAM1 expression on the endothelium and limits the ability of T cells to access the tumor, such that inhibition of ET_B_R improves T cell infiltration in an ICAM1-dependent manner (91). Others have shown that expression of Fas ligand (FasL) on the endothelium restricts leukocyte extravasation across the vascular barrier, including CD8+ T cells (92) and mononuclear cells (93), such that targeting FasL reverses this effect. These studies suggest that broadly targeting mechanisms of transmigration in the context of cancer would unlikely yield positive benefits; although this may reduce tumor cell extravasation, it may also restrict the infiltration of anti-tumor immune cells.

### Vascular Integrity and Permeability

Enhanced vascular permeability through downregulation of endothelial adhesions has been shown to influence the ease of tumor cell transmigration. While this can be regulated by a number of different factors, innate immune cells that are upregulated in response to tumor progression appear to play an important role. During tumor progression, macrophages, neutrophils, and various other myeloid cell types accumulate in both the primary tumor microenvironment and secondary niche. These cells contribute to a pro-inflammatory milieu that mimics normal responses to pathogen exposure, however, in the context of cancer, they can inadvertently facilitate dissemination (69). For example, in mouse models of breast cancer metastasis, CCR2+ inflammatory monocytes are attracted to the metastatic microenvironment by CCL2-producing tumor cells, where they promote vascular permeability and extravasation in a VEGFA-dependent manner (94). Tumor-derived CCL2 has also been shown to act directly on the endothelium to promote its activation, resulting in enhanced monocyte recruitment, dephosphorylation of VE-Cadherin, reduced tight junction integrity, and a consequential increase in tumor cell transmigration (95). Consistently, others have shown in mouse models of breast and lung cancer that inhibition of angiopoietin-2 (Ang2) (which is produced by the activated endothelium) in the post-surgical adjuvant setting improves pericyte coverage of the endothelium and reduces CCR2+ macrophage accumulation within secondary sites, leading to reduced metastatic progression (83). Therefore, the accumulation of inflammatory monocytes/macrophages that coincides with metastatic progression may dually serve to weaken endothelial barriers and enable additional tumor cells to access the metastatic niche. This may be in part due to the armamentarium of proteases that macrophages produce, which can cleave adhesions between endothelial cells. This has even been shown in mouse models of breast cancer metastasis through the blood-brain barrier, which is weakened by Cathepsin S production even though it should otherwise be a tight barricade to exclude peripheral cells and inflammatory factors from being able to access the brain parenchyma (96).

Neutrophils are another potent source of cytokines and proteases (most notably MMPs, NE, and cathepsin G) that can trigger vascular inflammation. This is an essential function so that neutrophils can rapidly access tissues as the first line of defense in the innate immune system (17). In mouse models of breast cancer metastasis, neutrophils promote metastasis by impairing the tumor-clearance capacity of NK cells in the circulation, and by releasing elevated levels of IL1β, MMP8, and MMP9 into the microenvironment, which increase vessel permeability (97). Additionally, in mouse models of melanoma and Lewis lung carcinoma, lung metastasis is enhanced in LPS-instilled lungs through the local recruitment of neutrophils, and their subsequent degranulation to release NE and cathepsin G (98). This causes protease-mediated degradation of the adhesion protein thrombospondin-1 and results in enhanced lung metastasis. ROS production by neutrophils has also been shown to promote tumor metastasis, through induction of neutrophil extracellular traps (NETs) (99–102); NETs may promote metastasis by trapping tumor cells (99) and/or by remodeling the extracellular matrix to awaken dormant tumor cells (102). ROS production by neutrophils has also been shown to promote tumor metastasis through the suppression of T cell immunosurveillance (103, 104). However, the role of neutrophil-ROS in vascular permeability during tumor metastasis specifically is less understood, despite its known role during inflammation. Therefore, cytokines, proteases and ROS that are produced by neutrophils to facilitate peripheral recruitment of immune cells during normal inflammatory responses may similarly facilitate peripheral recruitment of tumor cells. Thus, the ability of tumor cells to stimulate the accumulation and activation of neutrophils within the microenvironment (97, 98, 105–109) represents a critical way that tumors highjack and manipulate their niche to support their own progression.

Given the role of platelets during leukocyte extravasation, it is not surprising that they have similarly been shown to influence tumor cell extravasation. In murine models of experimental lung metastasis, platelet-tumor cell interactions promote tumor cell extravasation through the secretion of TGF-β from platelets and the subsequent activation of Smad and NFκB signaling within colon and breast carcinoma cells (110). This facilitates progression to an invasive mesenchymal-like phenotype and metastatic progression. Platelets have also been shown to recruit granulocytes to colon tumor cells within the lung in murine models of experimental lung metastasis, allowing for the formation of “early metastatic niches” in the lung microenvironment (111). Furthermore, using both *in vitro* Transwell assays and murine spontaneous lung metastasis assays, platelets activated by melanoma or lung tumor cells facilitated tumor cell transendothelial migration and extravasation via the secretion of adenine nucleotides (112). This promoted the opening of the endothelial barrier by acting on the endothelial P2Y_2_ receptor, supporting metastasis. Thus, platelets can modulate tumor cells, innate immune cells and/or the endothelium to facilitate the metastatic process.

## Targeting Innate Immunity to Improve Vascular Integrity as Cancer Therapy

There are several therapeutic approaches that may be useful to minimize chronic vascular inflammation and thus impede the ability of tumor cells to access the metastatic niche. One obvious approach is to target the vasculature directly, for example through anti-angiogenic strategies like bevacizumab (a VEGFA neutralizing antibody). However, while preclinical studies using anti-VEGFA antibodies showed great success leading to their clinical development (113), bevacizumab only improved progression-free survival, but not overall survival, in clinical trials for metastatic breast cancer (114–117). Although limiting nutrient delivery to tumors may seem logical to restrict viability and growth, crude attempts to broadly ablate the tumor vasculature may mitigate the beneficial effects of the blood vessels, such as leukocyte infiltration, oxygenation, and drug delivery. Vascular normalization strategies that aim to improve vascular maturation and integrity have been proposed as an alternative to anti-VEGFA treatments (118). For instance, preclinical studies have shown that VEGFR2 antibody blockade using DC101 can normalize the structure of the tumor-associated endothelium by improving the quality of the basement membrane and enabling improved pericyte coverage (119, 120). Whether these normalization strategies will be effective in the context of metastatic cancer, and how this will influence tumor cell interactions with the endothelium, have yet to be determined.

Given the potentially beneficial effects of luminal adhesions in bringing specific types of immune cells into tumors to enhance anti-tumor immunity, disrupting endothelial cells broadly may not be an optimal therapeutic approach. Several methods to enhance anti-tumor lymphocyte-specific recruitment have been proposed (121). For example, in ovarian cancer patients, ET_B_R expression correlates with low tumor infiltrating lymphocytes, and experimental models have shown that pharmacological blockade of ET_B_R with BQ-788 enhances T cell infiltration into tumors by modifying the endothelial barrier via a NO- and ICAM1-dependent mechanism (91). Importantly, rendering tumors “immune hot” through this method enhanced response to immunotherapy via cancer vaccination, whereas control tumors remained unresponsive (91). Interestingly, studies have also shown that VEGFA induces the expression of luminal adhesion proteins on endothelial cells, including ICAM1, VCAM1, and E-selectin, and that this can be blocked using an NFκB inhibitor, pyrrolidine dithiocarbamate (PDTC) (122), a chemical compound that dually serves as an oxygen radical scavenger. If these adhesions play a functional role in anti-tumor lymphocyte recruitment, this may partially contribute to the limited effects of anti-VEGFA therapies. Together these studies and others support the notion that endothelial barrier phenotypes and immune-surveillance are two intimately linked components of an immunoregulatory program in cancer, and that reprogramming the endothelium to enable leukocyte entry into tumors may have beneficial anti-tumor effects (123). This becomes particularly relevant in the context of cancer immunotherapy, as “immune-hot” tumors (i.e. those with high abundance of tumor-infiltrating lymphocytes) are more likely to respond to immune checkpoint blockade. Of note, the endothelium itself is capable of expressing checkpoint molecules that can negatively regulate T cell responses, including PDL1, PDL2, and TIM3 (124–126); whether endothelial-specific expression of these factors functionally influences response to immune checkpoint blockade remains uncertain.

Alternatively, there may be therapeutic opportunities to target innate immune cells in the microenvironment that both regulate vascular phenotypes, and dually act on tumor cells directly to promote progression. For example, several studies have shown that neutrophil depletion through antibody blockade can reverse metastasis of breast cancer (106, 108), including in the experimental setting where metastasis is assessed after 48 h following tail vein injection (potentially representative of extravasation) (97, 127). Alternatively, pharmacologic inhibition of CXCR2 has also been explored as a therapeutic approach to limit neutrophil infiltration and improve T cell infiltration in association with reduced metastatic progression in pancreatic models (128), which may help mitigate chronic oxidative and proteolytic effects on the endothelium. Indeed, pharmacologic agents targeting CXCR2 such as AZD5069 are now being explored in the clinical setting for metastatic cancer. In addition, Tie2-expressing monocytes/macrophages can trigger angiogenesis and vascular activation by inducing the expression of ICAM1 on the endothelium through interactions with its ligand, Ang2 (83, 129, 130), and several compounds that inhibit the Ang2-Tie2 axis are now being explored in the clinical setting for metastatic cancer including in the context of improving response to immune checkpoint inhibitors (131). Taken together, these trials demonstrate the clinical relevance of targeting vascular inflammation in cancer patients to improve metastatic outcomes.

## Conclusions and Future Perspectives

Innate immunity and vascular inflammation are two intimately connected biological processes that rely on one another to mediate physiologic responses to infection/inflammation. However, these intricate networks become undone in the context of cancer, and can be amplified by chronic inflammatory states. Given the complex nature of cell-cell interactions within the tumor microenvironment, consideration of all cellular players during different stages of the metastatic cascade is critical in order to optimize disease outcomes. Broadly inhibiting specific cell types is unlikely to yield favorable benefits; rather, reprogramming the microenvironment to work favorably and productively is key to improving survival. The endothelium in particular regulates multifaceted aspects of the microenvironmental landscape in all tissues throughout the body, as it is the gatekeeper of immune cell transmigration, nutrient and oxygen delivery, and a critical source of systemic soluble factors. Cancer hijacks these critical roles, and takes advantage of vascular plasticity to support disease progression. Therefore, by improving our understanding of normal physiologic functions of blood vessels and their interactions with regulatory cells within their environment, we will be able to improve our ability to target specific aspects of extravasation and metastasis by reprogramming the microenvironment to our advantage.

## Author Contributions

SM and DQ reviewed the literature and wrote the manuscript.

### Conflict of Interest Statement

The authors declare that the research was conducted in the absence of any commercial or financial relationships that could be construed as a potential conflict of interest.
